# T-Cell-Specific Loss of the PI-3-Kinase p110α Catalytic Subunit Results in Enhanced Cytokine Production and Antitumor Response

**DOI:** 10.3389/fimmu.2018.00332

**Published:** 2018-02-27

**Authors:** Laura Aragoneses-Fenoll, Gloria Ojeda, María Montes-Casado, Yeny Acosta-Ampudia, Umberto Dianzani, Pilar Portolés, José M. Rojo

**Affiliations:** ^1^Unidad de Inmunología Celular, Centro Nacional de Microbiología, Instituto de Salud Carlos III, Majadahonda, Madrid, Spain; ^2^Departamento de Medicina Celular y Molecular, Centro de Investigaciones Biológicas, CSIC, Madrid, Spain; ^3^Interdisciplinary Research Center of Autoimmune Diseases (IRCAD), Department of Health Sciences, University of Piemonte Orientale (UPO), Novara, Italy

**Keywords:** PI3K, PI3-kinase alpha subunit, T lymphocytes, CD28 costimulation, anti-KLH response, melanoma

## Abstract

Class IA phosphatidylinositol 3-kinase (PI3K) catalytic subunits p110α and p110δ are targets in cancer therapy expressed at high levels in T lymphocytes. The role of p110δ PI3K in normal or pathological immune responses is well established, yet the importance of p110α subunits in T cell-dependent immune responses is not clear. To address this problem, mice with p110α conditionally deleted in CD4^+^ and CD8^+^ T lymphocytes (p110α^−/−^ΔT) were used. p110α^−/−^ΔT mice show normal development of T cell subsets, but slightly reduced numbers of CD4^+^ T cells in the spleen. “*In vitro*,” TCR/CD3 plus CD28 activation of naive CD4^+^ and CD8^+^ p110α^−/−^ΔT T cells showed enhanced effector function, particularly IFN-γ secretion, T-bet induction, and Akt, Erk, or P38 activation. Tfh derived from p110α^−/−^ΔT cells also have enhanced responses when compared to normal mice, and IL-2 expanded p110α^−/−^ΔT CD8^+^ T cells had enhanced levels of LAMP-1 and Granzyme B. By contrast, the expansion of p110α^−/−^ΔT iTreg cells was diminished. Also, p110α^−/−^ΔT mice had enhanced anti-keyhole limpet hemocyanin (KLH) IFN-γ, or IL-4 responses and IgG1 and IgG2b anti-KLH antibodies, using CFA or Alum as adjuvant, respectively. When compared to WT mice, p110α^−/−^ΔT mice inoculated with B16.F10 melanoma showed delayed tumor progression. The percentage of CD8^+^ T lymphocytes was higher and the percentage of Treg cells lower in the spleen of tumor-bearing p110α^−/−^ΔT mice. Also, IFN-γ production in tumor antigen-activated spleen cells was enhanced. Thus, PI3K p110α plays a significant role in antigen activation and differentiation of CD4^+^ and CD8^+^ T lymphocytes modulating antitumor immunity.

## Introduction

Phosphoinositide-3 kinases (PI3K) phosphorylate inositol-containing lipids (PtdIns) at the D3 OH– group of the inositol ring. Generation of PtdIns(3)P in the inner leaflet of membrane bilayers allow the recruitment of cytosolic proteins that participate in distinct signaling cascades [reviewed in Ref (1–5)]. Three different classes of PI3K (class I, class II, and class III PI3K) are grouped according to their structure, regulation of activity, and preference for lipid substrate. Among these, the activity of class I PI3K that phosphorylate PtdIns(4,5)P_2_ yielding PtdIns(3,4,5)P_3_ is frequently altered in cancer cells and are important targets in cancer therapy (6–8).

Class IA PI3-kinases are heterodimers of one catalytic subunit (p110α, p110β, or p110δ) and one regulatory subunit (p85α, p55α, p50α, derived from the same gene by alternative splicing, or p85β) that characteristically bind to proteins phosphorylated at the tyrosine of YxxM motifs (1, 4, 5), including molecules of the CD28 family (9–12). Class IB PI3-kinases comprise one p101 or p84/87^PIKAB^ regulatory subunit and one p110γ catalytic subunit. Class IB, but also class IA p110β kinases, are recruited and activated upon ligand binding to cell surface G-protein coupled receptors (1, 4, 5). Whereas p110α and p110β catalytic subunits have a wide tissue distribution, p110γ and p110δ subunits are mainly expressed by cells of hematopoietic origin. Genetic and pharmacological data show that these Class I PI3-kinases are of prime importance to lymphocyte differentiation and activation necessary to develop effective immune responses (2, 4, 13–15). Thus, the analysis of PI3-kinases in immune cells has focused on these subunits, as potential targets for immune intervention. Indeed, there is abundant evidence of the importance of both p110γ and p110δ in different aspects of normal or pathological leukocyte function and immune reactions [Reviewed in Ref (2, 4, 13, 16, 17)].

The abundant expression of class IA p110δ catalytic subunits by leukocytes, and the functional association of p110δ to the antigen receptors of T and B lymphocytes might point to these catalytic subunits as the main source of class IA PI3-kinase activity in lymphocytes (13, 18, 19). Indeed, genetic and pharmacological data confirm the importance of p110δ to B and T cell antigen activation, and p110δ-specific inhibitors can induce inhibition of TCR activation, or costimulation by YxxM motif-containing molecules like CD28 or ICOS (20–22).

We have previously shown that PI3-kinase p110α catalytic subunits are expressed by CD4^+^ T lymphocytes, or T cell lines at levels comparable to those of p110δ subunits (20). This is also found in other leukocyte populations (23). Interestingly, p110α subunits were preferentially recruited to phosphorylated ICOS or CD28 (20); this was due to the preferential association of p110α catalytic subunits to the regulatory subunits of class IA PI-3 kinases that bind to their phosphorylated YxxM motifs. In agreement with these data, inhibition or silencing p110α blocked ICOS-dependent elongation or costimulation of early TCR signals and cytokine secretion in T cell lines (20).

Here, we have used T-lymphocyte-specific deletion of p110α PI3-kinase to further analyze the role of p110α in T cell activation, CD28-dependent costimulation, and T-dependent responses. Our data show that CD4^+^ and CD8^+^ T lymphocytes lacking p110α (p110α^−/−^ΔT) have enhanced effector function and hampered expansion of Treg cells. Particularly, they show higher IFN-γ secretion *in vitro* that correlate with higher T-bet levels and early Erk and P38 activation. Using protein antigens and experimental models of melanoma we show that p110α^−/−^ΔT mice have altered humoral responses and inhibited growth of melanoma linked to enhanced antigen-specific IFN-γ responses and lower Treg cell numbers.

## Results

### Activation of PI3-Kinase p110α-Deficient CD4^+^ T Cells

PI3-kinase p110α-null mice are embryonic lethal (24). So, to assess the role of p110α PI3-kinase in T cell function, mice with conditional deletion of the *pik3ca* gene in T cells were generated by crossing CD4-Cre mice and mice with a floxed *pik3ca* gene (p110α^flox/flox^) (24). CD4-Cre^+/−^/p110α^flox/flox^ mice will be hereinafter referred to as p110α^−/−^ΔT, whereas CD4-Cre^−/−^/p110α^flox/flox^ littermates will be termed wild type (WT). PI3-kinase p110α was efficiently removed from peripheral CD4^+^ and CD8^+^ T lymphocytes of p110α^−/−^ΔT mice; however, the PI3-kinase p110δ subunit or other proteins like CD4, or Erk were unaffected (see Figure S1 in Supplementary Material, and data not shown). Most subpopulations in the peripheral lymphoid organs of WT and p110α^−/−^ΔT mice were not significantly changed, including the percentage of total T (CD3^+^) cells, CD8 T lymphocytes, B lymphocytes, γδ, and NKT lymphocytes, or NK cells (see Figure S1 in Supplementary Material, and data not shown). However, p110α^−/−^ΔT mice showed a slightly lower number of spleen cells and a lower percentage of CD4^+^ cells (Figure S1 in Supplementary Material), even though the percentage of naive and memory or Treg cells within the CD4^+^ T cell population was not significantly different. Analysis of thymus cells indicated that this was not due to a deficient development of mature CD4^+^ T lymphocytes (Figure S1 in Supplementary Material).

**Figure 1:**
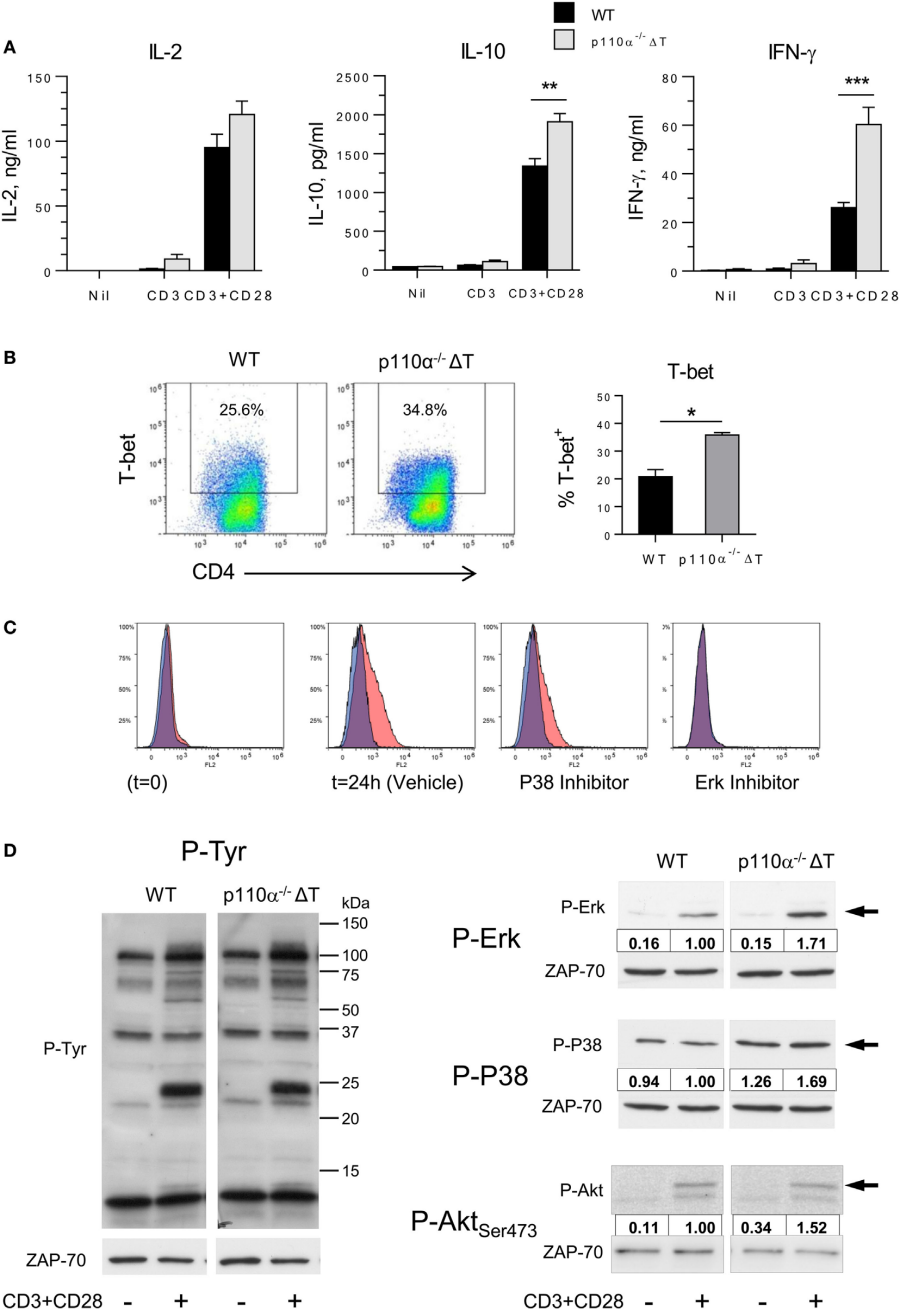
Effect of PI3-kinase p110α removal on naive T cell activation. **(A)** Naive CD4^+^ T lymphocytes from WT or p110α-T cell deficient (p110α^−/−^ΔT) mice were activated with plate-bound anti-CD3 plus anti-CD28, as indicated. At 72 h, IL-2, IL-10, and IFN-γ content in the supernatants was determined. Mean from three experiments ± SE. Asterisks indicate significant differences (**p < 0.01; ***p < 0.001) between groups, as determined by the Student’s *t*-test. **(B)** Naive CD4^+^ T lymphocytes from WT or p110α^−/−^ΔT mice (WT) were activated with plate-bound anti-CD3 plus anti-CD28 for 24 h, then analyzed for T-bet expression by flow cytometry. Mean from three determinations ± SE. Significant differences between data are indicated (*p < 0.05). **(C)** Effect of MAPK inhibitors on T-bet induction. Naive CD4^+^ T lymphocytes from WT mice were activated as in **(B)** in the presence of vehicle (DMSO), or the P38 inhibitor SB203580 (2 µM), or Erk inhibitor U0126 (5 µM), as indicated in the histograms. Red histograms: anti-T-bet; Blue histograms: control antibody. **(D)** WT or p110α^−/−^ΔT naive CD4^+^ T cells were activated for 20 min with CD3 plus CD28 stimulus. Cell lysates were then analyzed by immunoblot. Cells lacking PI3K p110α show increased phosphorylation of distinct proteins (left panel) as well as of activated forms of the ERK and P38 MAP kinases (center and right panels, and histograms), or Akt Ser_473_ phosphorylation (right panels). Relative OD of CD3/CD28-activated WT cells was considered = 1.00. Data from one experiment of three independent experiments performed.

Next, the effect of PI3-kinase p110α removal on the activation of naive CD4^+^ T lymphocytes *in vitro* was determined. Secretion of IL-10 and, particularly, IFN-γ were significantly enhanced in p110α^−/−^ΔT cells activated with anti-CD3 plus anti-CD28 antibodies, as compared to WT littermates (Figure 1A). In contrast, IL-2 secretion or proliferation was not significantly changed (Figure 1A, and data not shown). The levels of the IFN-γ master transcription factor T-bet were also significantly enhanced in activated CD4^+^ T cells of p110α^−/−^ΔT mice (Figure 1B). Induction of T-bet expression in CD4^+^ T lymphocytes depends on the activity of MAP kinases like P38 and, particularly, Erk, as revealed using specific inhibitors (Figure 1C). Consequently, the impact of p110α removal in early MAP kinase activation was analyzed (Figure 1D). As expected, activation of naive CD4^+^ T with anti-CD3 plus anti-CD28 induced Tyr phosphorylation of specific substrates, and some of them showed enhanced phosphorylation in p110α^−/−^ΔT cell lysates. Furthermore, Erk activation was clearly higher in p110α^−/−^ΔT cells than in WT cells. In unstimulated WT cells, the basal phosphorylation of P38 was not significantly changed upon anti-CD3 plus anti-CD28 stimulation. In p110α^−/−^ΔT cells, the basal level of P38 activation was higher than in WT cells, and was enhanced by CD3 plus CD28 stimuli (Figure 1D). Interestingly, T cells lacking p110α show enhanced levels of phosphorylation of the PI3K target Akt. This suggests that other PI3K catalytic subunits like p110δ can advantageously replace p110α concerning PI3K activation. Taken together, these data indicate that p110α removal enhances early activation signals in CD4^+^ T lymphocytes, leading to enhanced MAPK activity, T-bet induction, and eventually to higher IFN-γ secretion.

### Effect of PI3-Kinase p110α-Deficiency on the Differentiation of Effector CD4^+^ T Cells

Naive CD4^+^ T cells were then differentiated into effector T cell populations “*in vitro*,” as under neutral activation conditions, i.e., in the absence of added cytokines, the effect of p110α-deficiency on cytokines other than IL-2, IL-10, or IFN-γ could not be determined.

**Figure 2:**
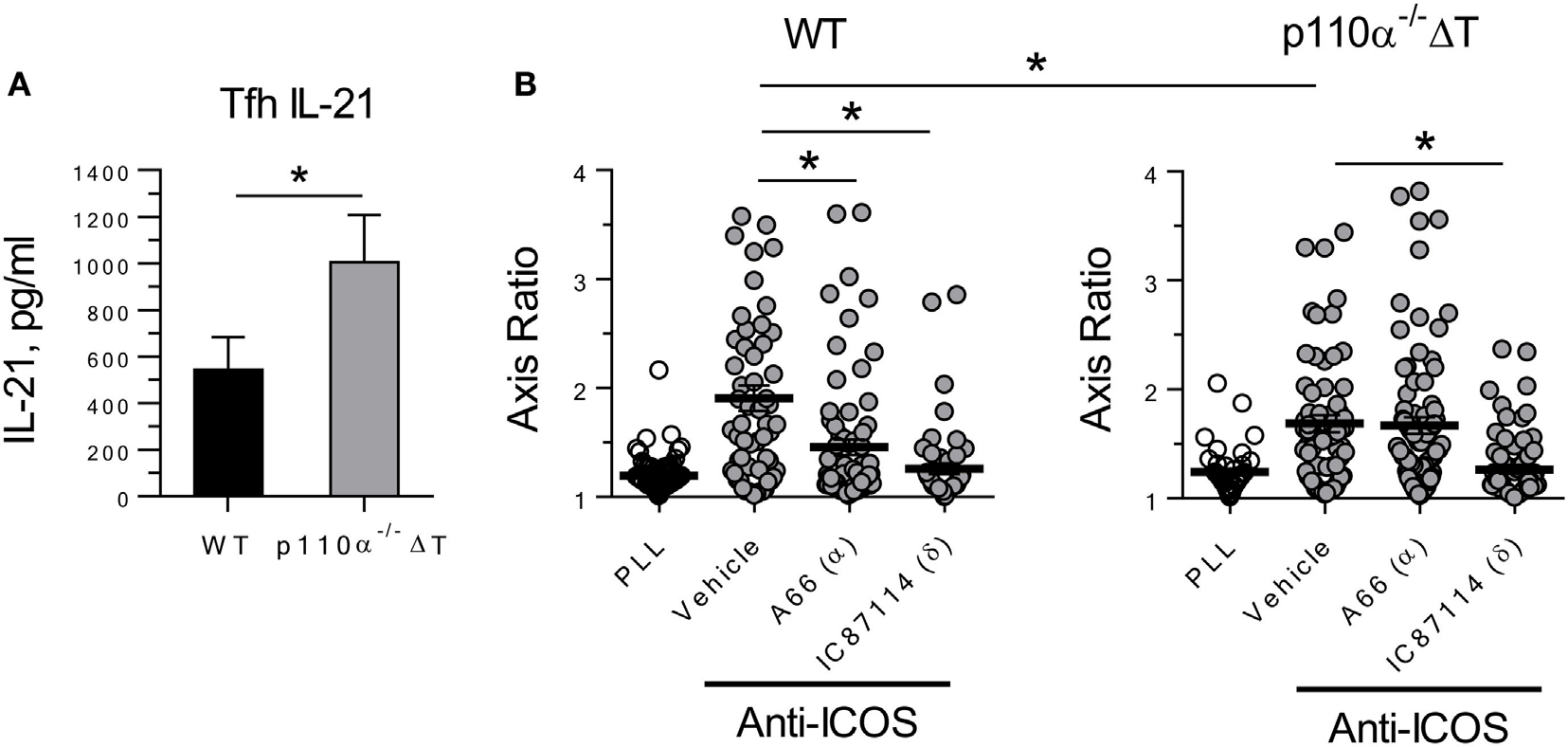
PI3-kinase 110α-deficient Tfh cells show enhanced secretion of the effector cytokine IL-21 **(A)**, but partially impaired ICOS-induced elongation **(B)**. Naive CD4^+^ T lymphocytes from WT or p110α-T cell deficient (p110α^−/−^ΔT) mice were differentiated for 4 days “*in vitro*” with anti-CD3 plus anti-CD28 antibodies under Tfh conditions. Then, culture supernatants were analyzed for IL-21 **(A)**, or ICOS-induced elongation of Tfh cells was determined **(B)**, as indicated. Mean ± SE of four experiments **(A)**. In **(B)**, the elongation of WT or p110α-T cell-deficient Tfh cells was determined as the axis ratio in the presence of plate-bound Poly-L-Lys (PLL) or anti-ICOS, as indicated. The cultures received vehicle (DMSO), or inhibitors specific for PI3-kinase p110α (A66, 1 µM) and p110δ (IC87114, 5 µM). Mean ± SE of elongation in >50 individual cells. Significant differences (*p < 0.05; ***p < 0.001) were determined using the Student’s *t*-test.

In differentiated Tfh cells, the production of IL-21 (Tfh) was significantly enhanced in p110α-deficient cells (Figure 2A). Then, ICOS-induced elongation of Tfh cells was analyzed. Tfh express ICOS at high levels, and it is known that ICOS interaction with ICOS-ligand expressed by bystander B cells is needed for Tfh cells to reach germinal centers. This phenomenon is dependent on PI3-kinase activity, so that ICOS-induced, PI3-kinase-dependent elongation of ICOS^+^ T cells might be involved in germinal center formation *in vivo*. Our previous data using specific inhibitors indicated that, in Th2 cell lines, ICOS-induced elongation is largely dependent on the activity of the p110α PI3K subunits (20). Hence, anti-ICOS antibody was used to compare the elongation of “*in vitro*” differentiated Tfh cells of p110α^−/−^ΔT or WT genotype. As shown in Figure 2B, the contribution of p110α to this phenomenon was confirmed by the significantly lower elongation of p110α^−/−^ΔT Tfh cells. Indeed, the elongation of Tfh cells from WT, but not p110α^−/−^ΔT mice was significantly inhibited by a p110α-specific PI3K inhibitor (A66, 1 µM). In both cases, a p110δ-specific inhibitor (IC87114, 5 µM) blocked ICOS-induced elongation.

Our results using Th1 T cells differentiated “*in vitro*” indicated that IL-2 and IFN-γ secretion as well as T-bet expression were enhanced in cells lacking PI3K p110α (Figure S2A in Supplementary Material), in agreement with the results obtained under neutral conditions (Figure 1). In differentiated Th17, the production of IL-17A was also enhanced in p110α-deficient cells Figure S2A in Supplementary Material.

**Figure 3:**
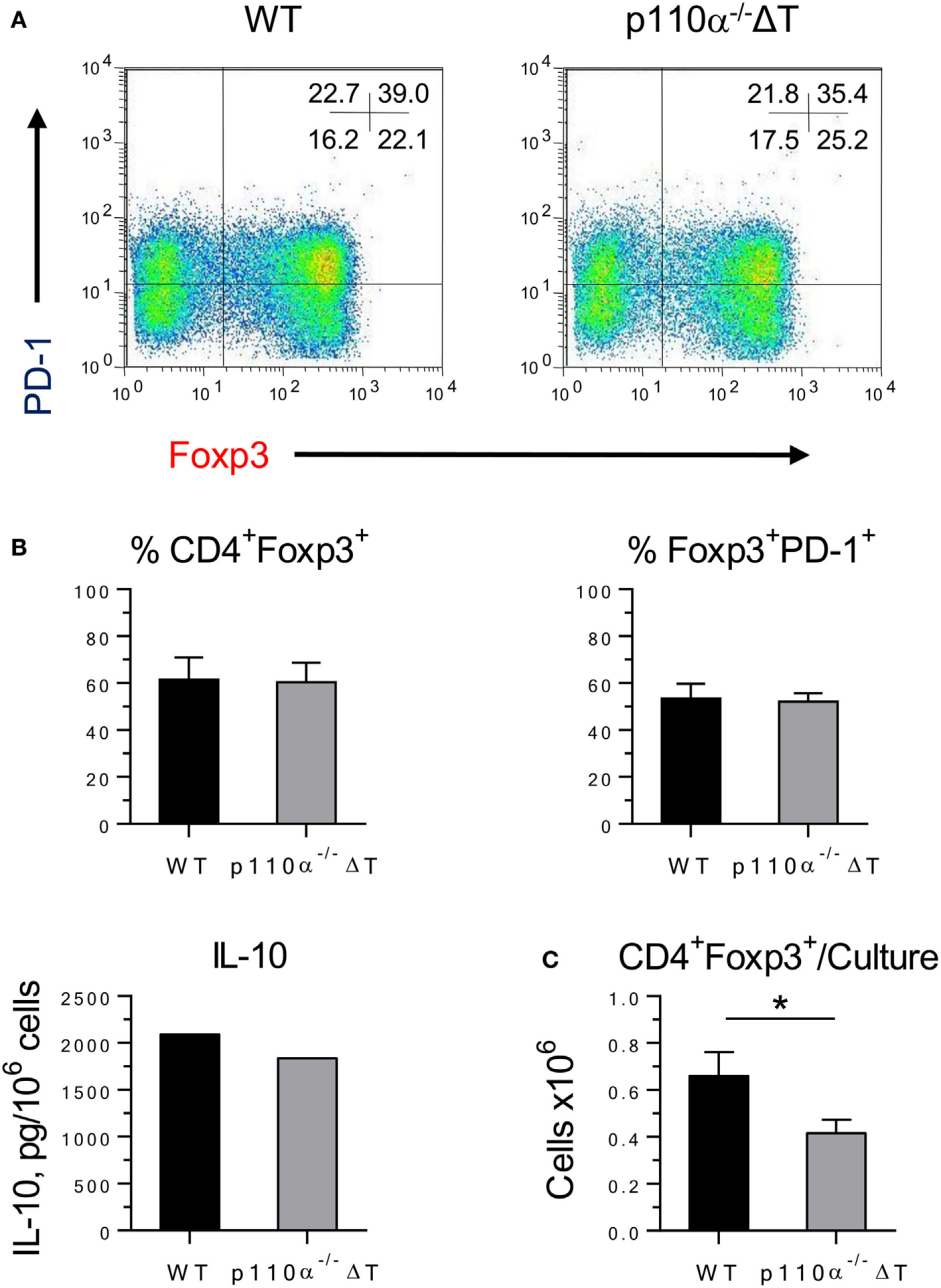
Differentiation of iTreg in WT or PI3-kinase p110α deficient (p110α^−/−^ΔT) CD4^+^ T cells. **(A)** Naive CD4^+^ T lymphocytes were cultured for 48 h under conditions favoring Treg differentiation. The percentage of iTreg (CD4^+^Foxp3^+^) cells and the expression of PD-1 were analyzed by flow cytometry. Figures inside the histograms represent the percentage of cells in each quadrant. Data from one representative experiment of four performed. **(B)** Percentage of iTreg (Foxp3^+^ cells in CD4^+^ cells); PD-1^+^ cells in Foxp3^+^ cells; and IL-10 in culture supernatants. **(C)** Number of iTreg cells/culture in naive CD4^+^ T lymphocytes from WT or p110α^−/−^ΔT mice cultured under Treg conditions. Mean from four experiments ± SE. IL-10, mean from two experiments with similar results. Asterisks indicate significant differences (*p < 0.05) between groups, as determined by the Student’s *t*-test.

Last, the differentiation of naive CD4^+^ T cells into iTreg was checked. No significant differences were observed in the percentage of CD4^+^Foxp3^+^ (Figure 3), of Foxp3^+^ cells expressing the inhibitory checkpoint marker PD-1 (Figure 3), or of Foxp3^+^ cells expressing ICOS (data not shown). Furthermore, similar amounts of IL-10 were secreted on a per-cell basis (Figure 3B). In contrast, the number of cells recovered from p110α^−/−^ΔT cultures was significantly lower than from WT cultures, and so were the number of iTreg cells (Figure 3C). Overall, this might result in a weaker Treg activity in p110α^−/−^ΔT mice.

### p110α Deficiency Enhances CD8^+^ T Cell Function

**Figure 4:**
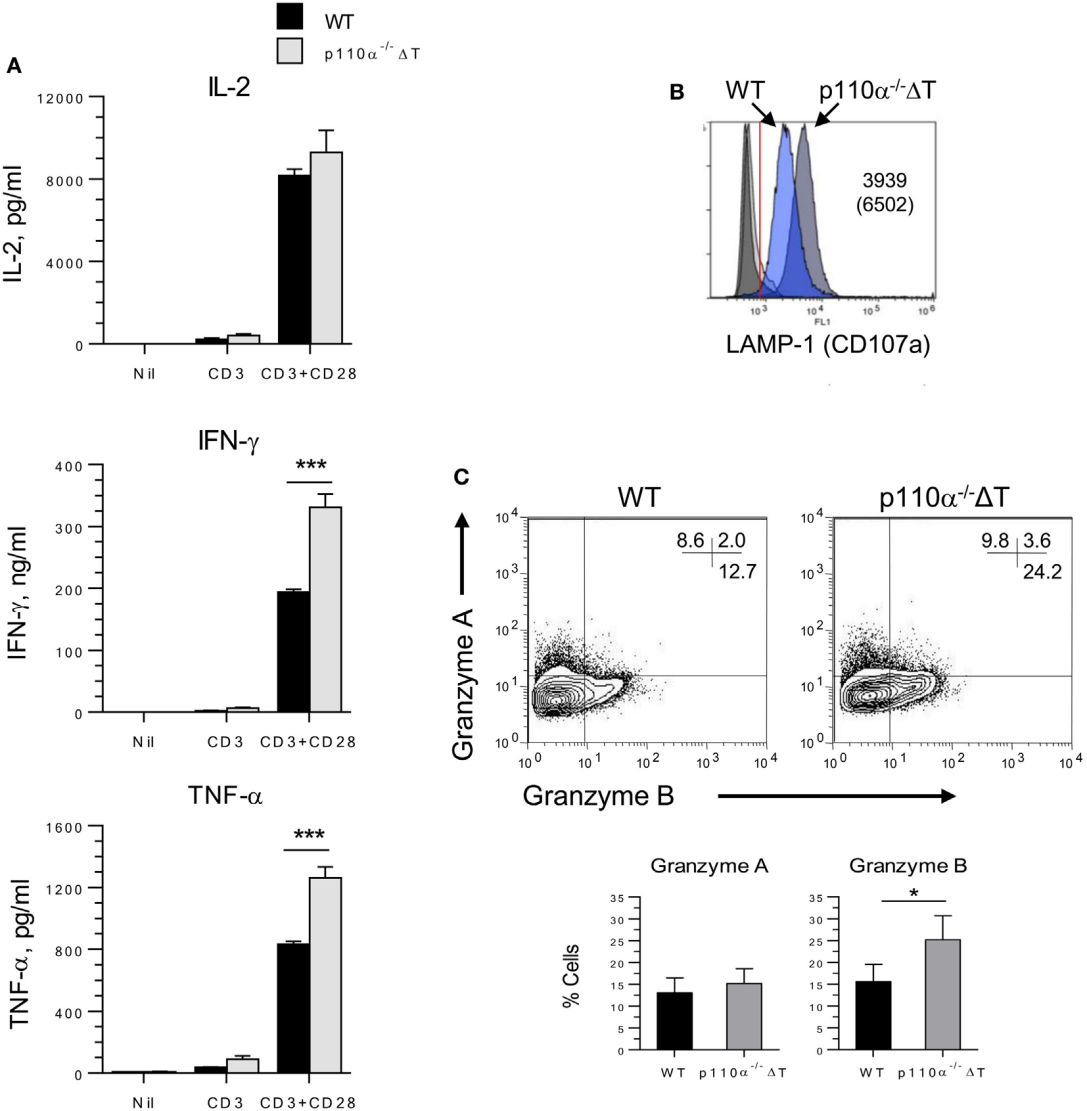
PI3-kinase p110α deficiency enhances activation and effector functions of CD8^+^ naive T cells. **(A)** Naive (CD8^+^ CD62L^+^) CD8^+^ T lymphocytes were isolated from the spleen of WT or p110α^−/−^ΔT mice. They were activated for 3 days with anti-CD3 and anti-CD28, as shown in the figure. Then, the culture supernatants were taken and analyzed for IL-2, IFN-γ, and TNF-α content. Data from three experiments ± SE. Significant differences (***p < 0.001) between groups are indicated, as determined by the Student’s *t*-test, n.s., not significant. Alternatively, CD8^+^ T cells were activated 72 h with anti-CD3 plus anti-CD28 antibodies, washed, and cultured 72 h in the presence of IL-2. Then, the expression in the expanded cells of LAMP-1 **(B)**, or Granzymes A and B **(C)** was analyzed by flow cytometry. Mean ± SE from three independent experiments (Granzyme A, B). Significant differences (*p < 0.05; ***p < 0.001) between means are indicated, as determined by the Student’s *t*-test.

Then, the role of PI3K p110α deficiency on the activation and differentiation “*in vitro*” of T CD8^+^ lymphocytes was also addressed. Naive CD8^+^ T cells were cultured for 72 h with anti-CD3 and anti-CD28. IFN-γ levels were much higher in p110α^−/−^ΔT cell cultures, in agreement with the results observed in CD4^+^ T cells. As shown in Figure 4A, the levels of secreted TNF-α were clearly enhanced in activated p110α^−/−^ΔT cells, whereas IL-2 secretion was not significantly different in WT or p110α^−/−^ΔT cells.

Class I PI3-kinases control the differentiation of TCR-activated CD8^+^ in the presence of IL-2. So, naive CD8^+^ T cells were activated for 72 h with anti-CD3 and anti-CD28, washed, and expanded in the presence of IL-2 for further 72 h. The expanded p110α^−/−^ΔT cells also showed enhanced effector mechanisms. These included the expression of molecules involved in cytotoxic mechanisms like CD107a (LAMP-1) and Granzyme B (Figures 4B,C), or IFN-γ and TNF-α secretion (Figure S2B in Supplementary Material).

### Altered Immune Responses against Protein Antigens in Mice with p110α-Deficient T Cells

**Figure 5:**
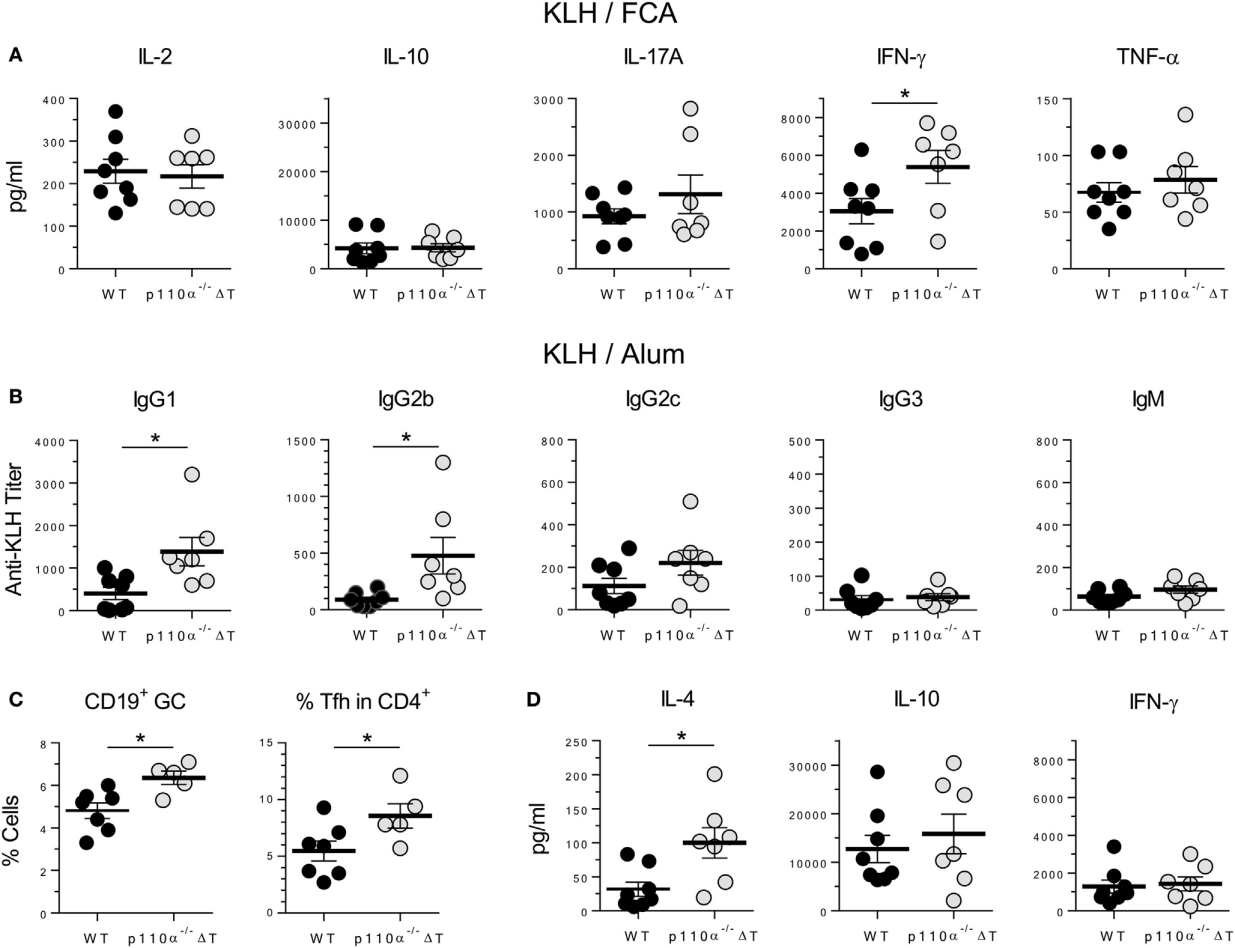
T-cell PI3-kinase p110α deficiency alters cytokine and antibody responses against the protein antigen keyhole limpet hemocyanin (KLH). **(A)** WT (*n* = 8) or p110α^−/−^ΔT mice (*n* = 7) were immunized s.c. with KLH plus FCA. Lymph node cells from individual mice were taken on day 7 after immunization, and cultured “*in vitro*” for further 96 h in the presence of KLH. Then, cytokines in the supernatant (IL-2, IL-10, IL-17A, IFN-γ, or TNF-α) were determined, as indicated in the figure. Individual values as well as means ± SE are shown. Data from two experiments. Significant differences (*p < 0.05) are indicated, as determined by the Student’s *t*-test. **(B)** KLH-specific response of WT or p110α^−/−^ΔT mice injected i.p. with KLH in Alum: antibodies of the IgG1, IgG2a, IgG2c, IgG3, and IgM isotypes were determined in the serum 10 days after immunization. **(C)** Percentage of germinal center B cells (CD19^+^PNA^+^) and Tfh cells (CD4^+^CXCR5^+^ICOS^+^) were determined in the spleen of KLH/Alum immunized mice. **(D)** Spleen cells from immunized mice were cultured “*in vitro*” for 96 h in the presence of KLH, and cytokines in the supernatant (IL-4, IL-10, IFN-γ) were determined. Data from one **(C)** or two experiments **(B,D)**. Individual values from *n* = 8 (WT) or *n* = 7 (p110α^−/−^ΔT) mice **(B,D)**, or *n* = 7 (WT) and *n* = 5 (p110α^−/−^ΔT) mice **(C)**, as well as means ± SE, are shown. Significant differences (*p < 0.05) are indicated, as determined by the Student’s *t*-test.

The impact of T cell PI3K p110α deficiency “*in vivo*” was also analyzed in a model of immune response to the protein antigen keyhole limpet hemocyanin (KLH). Mice were first immunized s.c. with KLH in Freund’s adjuvant (KLH/FCA). Cytokine release upon antigen re-stimulation “*in vitro*” of cells from draining lymph nodes was then determined (Figure 5A). The levels of IFN-γ in cell cultures from p110α^−/−^ΔT mice were significantly higher than those of WT mice. No significant differences were observed in the other cytokines examined (IL-2, IL-10, IL-17A, and TNF-α), even though the levels of the inflammatory cytokines IL-17A and TNF-α were higher in p110α^−/−^ΔT cells (Figure 5A). No significant differences were found in the levels of KLH-specific antibodies of the isotypes examined (Figure S3A in Supplementary Material).

Mice were also immunized i.p. with KLH and Alum as adjuvant (KLH/Alum). In these conditions, the level of anti-KLH-specific antibodies of the IgG1 and IgG2b isotypes was significantly higher in the sera of p110α^−/−^ΔT mice (Figure 5B), as was the percentage of germinal center B lymphocytes and T lymphocytes with a Tfh cell phenotype in the spleen cells from immunized mice (Figure 5C). Spleen cells were re-stimulated “*in vitro*” with KLH and the cytokines released determined (Figure 5D; Figure S3B in Supplementary Material). Significantly enhanced secretion of the Th2 cytokine IL-4 fitted with the higher titer of IgG1 anti-KLH antibodies, but no significant differences were observed in IL-10 and IFN-γ levels (Figure 5D) or in IL-2, IL-17A, and TNF-α levels (Figure S3B in Supplementary Material).

### p110α^−/−^ΔT Mice Show Enhanced Resistance to B16 Melanoma

**Figure 6:**
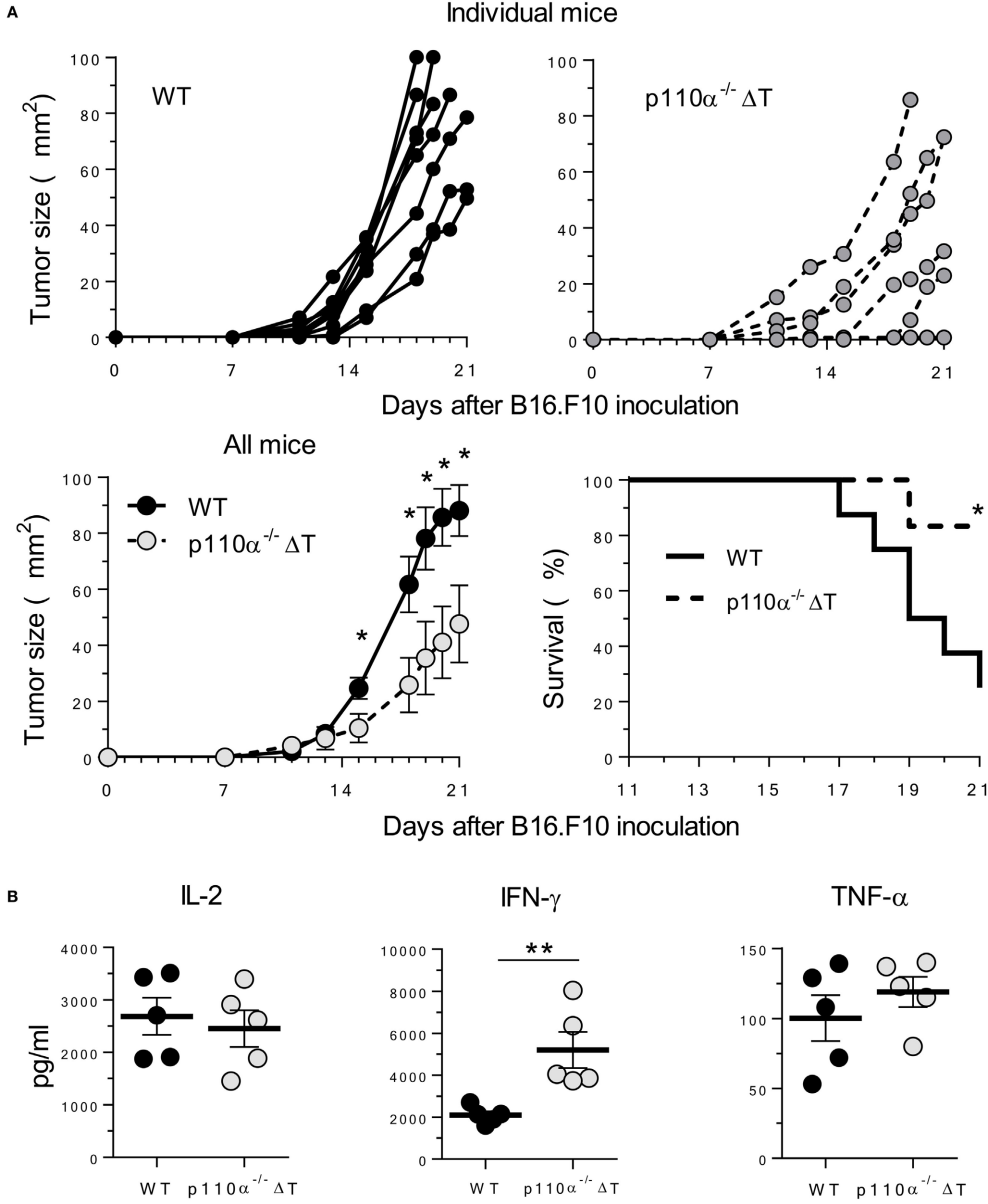

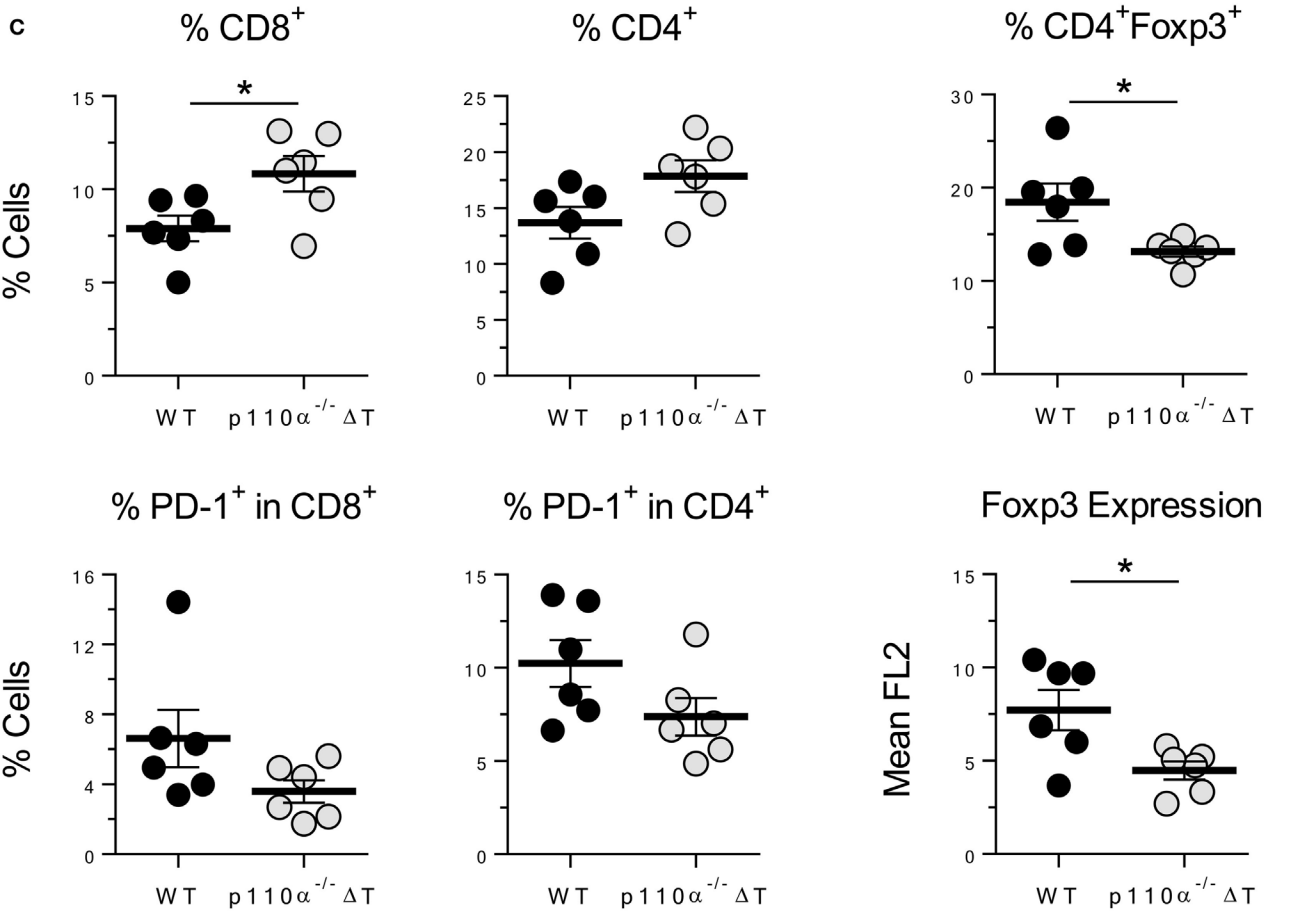
PI3-kinase p110α-deficiency in T cells inhibits the growth of B16.F10 melanoma cells. **(A)** B16.F10 melanoma cells were injected s.c. in WT or p110α-T cell deficient (p110α^−/−^ΔT) mice. Then, tumor growth and survival of mice were determined, as indicated. Data from two experiments are depicted as tumor size in individual mice (top panels) or as mean ± SE from *n* = 8 (WT) or *n* = 6 (p110α^−/−^ΔT) mice. Significant differences (*p < 0.05) are indicated, as determined by the Student’s *t*-test (lower left) or the long-rank Mantel–Cox test (lower right). **(B)** Spleen cells from melanoma-bearing mice were activated “*in vitro*” with melanoma proteins for 96 h. Then, cytokines in the supernatant (IL-2, IFN-γ, TNF-α) were determined. Data from two experiments. Individual values from *n* = 5 (WT) or *n* = 5 (p110α^−/−^ΔT) mice and means ± SE, are depicted. Significant differences are indicated (**p < 0.01), as determined by the Student’s *t*-test. **(C)** Expression of cell surface (CD4, CD8, PD-1) or intracellular markers (Foxp3) as determined by flow cytometry of spleen cells from WT (*n* = 6) or p110α^−/−^ΔT (*n* = 6) melanoma-bearing mice. Data expressed as percentage of cells or as mean fluorescence of Foxp3 in CD4^+^Foxp3^+^ cells (lower right panel). Individual values and mean ± SE are shown. Significant differences (* *p* < 0.01), as determined by the Student’s *t*-test, are indicated.

IFN-γ is a major mediator in antitumor adaptive responses. Since T cells from p110α^−/−^ΔT mice had enhanced effector mechanisms including IFN-γ secretion, and might have altered Treg proliferation, the effect of PI3K p110α deletion in T cells on the growth of s.c. injected B16.F10 melanoma was examined. Tumor growth was significantly lower in p110α^−/−^ΔT mice, as shown by tumor size or by the survival rate (Figure 6A). Furthermore, spleen cells from tumor-bearing mice were analyzed by flow cytometry and activated “*in vitro*” with melanoma proteins. Activated cells from p110α^−/−^ΔT mice secreted significantly higher amounts of IFN-γ; however, this difference was not found in other cytokines like IL-2 and TNF-α (Figure 6B). Interestingly, the percentage of CD8^+^ T cells was significantly enhanced in tumor-bearing p110α^−/−^ΔT mice (Figure 6C). Furthermore, CD4^+^Foxp3^+^ Treg cells (CD4^+^Foxp3^+^) were significantly diminished in p110α^−/−^ΔT mice, and the level of Foxp3 in these cells was lower than in their WT counterparts (Figure 6C). The fraction of CD4^+^ and CD8^+^ T lymphocytes expressing the inhibitory checkpoint marker PD-1 was also lower in the spleen of p110α^−/−^ΔT mice, yet the difference was not significant. In summary, our data show enhanced T-cell-mediated mechanisms of tumor rejection in p110α^−/−^ΔT mice.

## Discussion

Class IA PI3Ks are essential to normal development and function of adaptive immune reactions. In T and B lymphocytes, the p110δ isoform has a major role in ontogeny and adaptive immunity. Intriguingly, the p110α isoform is abundant in lymphocytes and can bind to and participate in the signals of costimulatory molecules like CD28 or ICOS (20, 25). Studies with isoform-specific inhibitors suggest that p110α might be relevant in certain T cell functions (i.e., IFN-γ or IL-21 production) but not in others (i.e., IL-2) (25, 26). Consequently, to further determine the importance of PI3K p110α in T cell function, CD4-Cre mice were crossed with mice with a floxed *pik3ca* gene (p110α^flox/flox^) to obtain mice (p110α^−/−^ΔT) whose T cells lacked the PI3K p110α isoform.

Previous data using CD2-Cre mice and p110α^flox/flox^ to delete PI3K p110α in lymphocytes indicated a role for p110α in pre-B cell receptor and tonic B-cell receptor signaling, contributing to B lymphocyte differentiation and B cell survival (27). In contrast, development and survival of CD3^+^ cells in these p110α-deficient mice was apparently unaffected (27). Indeed, we observed no significant differences in the thymus differentiation of T cells or in the proportion of total T (i.e., CD3^+^) lymphocytes in the spleen of p110α^−/−^ΔT animals. However, a closer analysis of lymphocyte subpopulations indicated a minor but significant decrease in the percentage of CD4^+^ T cells as well as in the number of spleen cells.

In the absence of additional cytokines or signals, CD4^+^ or CD8^+^ p110α^−/−^ΔT cells activated through TCR/CD3 and CD28 show augmented secretion of certain cytokines, particularly IFN-γ. Enhanced signaling is observed very early in the activation of naive T cells at the Tyr phosphorylation of cell substrates, or in the activation of Akt and the MAP kinases Erk and P38. Furthermore, the level of the Th1 master transcription factor T-bet is also higher in activated p110α^−/−^ΔT T cells.

Although these data suggest that Th1 responses are favored in the absence of p110α, our results also indicate that, depending on the conditions and/or the presence of additional cytokines, p110α^−/−^ΔT mouse T cells have significantly enhanced Tfh and effector CD8 cell responses “*in vitro*.” “*In vivo*,” injection of KLH in FCA induces enhanced IFN-γ responses, whereas immunization with the same antigen in Alum induces enhanced production of the Th2 cytokine IL-4. Although this suggests a widespread enhancing effect of p110α deletion, the absence of significant changes in some cases like IL-2 point to a selective effect on specific signal pathways and transcription factors.

Taken together, our results with p110α deletion, plus previous data using inhibitors suggest that p110α contribute to PI3K signaling upon T lymphocyte activation, but at the same time might compete with other catalytic PI3K subunits (i.e., p110δ) and down-modulate their signals. Since some molecules of the Ras family (including Ras) are activators of p110α but not p110δ (28) and are recruited by phosphorylated CD28 through GRB/Sos (29), it is possible that the activity of recruited Ras is directed to Raf/Erk activation rather than to PI3K activation when p110α is missing.

Changes in T lymphocyte activation and differentiation in p110α^−/−^ΔT mice have interesting consequences in antitumor immunity. When compared to WT mice, tumor-bearing p110α^−/−^ΔT mice have enhanced numbers of CD8^+^ T lymphocytes and enhanced production of IFN-γ. Furthermore, the percentage of Treg cells was lower in tumor-bearing p110α^−/−^ΔT mice. Overall, these features should delay melanoma growth. In our p110α^−/−^ΔT mice, p110δ is left as the main PI3K class IA subunit present in T lymphocytes. Interestingly, PI3K p110δ activity is necessary for efficient Treg expansion and function, and Tregs have a particular sensitivity to p110δ inactivation or inhibition (30–32). So, mice with inactive p110δ or p110δ-specific inhibitors favor antitumor immunity by preventing Treg cell-mediated suppression (32). However, there are also data indicating that activation of the PI3K/AKT/mTOR axis inhibits iTreg (33–37), and the p110α isoform might play a relevant role (34).

Our data show that Treg numbers are not significantly altered in p110α^−/−^ΔT mice under homeostatic conditions. Treg differentiation “*in vitro”* is also unaffected in p110α^−/−^ΔT, as far as the percentage of Foxp3^+^ cells, PD-1 expression, or IL-10 secretion is concerned; however, absolute numbers were lower in p110α^−/−^ΔT iTreg cultures. This effect was observed under different culture conditions provided TGF-β was present (data not shown), suggesting that, in our conditions, p110α is needed for iTreg expansion and/or survival *in vitro*. Whereas the percentage of Treg in the spleen of tumor-bearing mice was elevated compared to normal mice, p110α^−/−^ΔT tumor-bearing mice had significantly lower Treg than WT mice. Previous data showing that Akt/mTOR activation fosters Th1 but hamper TGF-β signaling and Treg differentiation (38) fit with the enhanced Th1 response and lower Treg numbers observed in p110α^−/−^ΔT T cells; this might well be due to overall enhancement of PI3K activity through p110δ signaling. Alternatively, p110α might participate in IL-2R PI3K signals and PTEN expression in Treg, as IL-2-induced expansion of Treg needs IL-2 activation of the PI3K/Akt axis, but also TCR signals that block PTEN expression (39). These possibilities need to be further ascertained.

In summary, our data indicate that the p110α PI3K catalytic subunit contributes to and regulates the antigen activation and differentiation of CD4^+^ and CD8^+^ T lymphocytes, including antitumor immunity. This points to the importance of assessing the impact in immune responses of anticancer therapies using PI3K p110α inhibitors, particularly when combined with immunotherapy (40), or the importance of p110α in autoimmune diseases. Interestingly, recent data indicate that CD28, and particularly CD28 recruitment of PI3K, is a major target of PD-1-mediated dephosphorylation and PD-1 targeted immunotherapy (41, 42).

## Materials and Methods

### Mice

Mice used in this study were C57BL/6J, CD4-Cre [(43) Tg(Cd4-cre)1Cwi; MGI:2386448, backcrossed for nine generations with C57BL/6NTac mice], and *Pik3ca^flox^*, p110α^flox/flox^ described in Ref. (24). They were bred under specific pathogen-free conditions in the animal care facility of the Centro de Investigaciones Biológicas from stock purchased from Charles River (C57BL/6J, p110α^flox/flox^), or the European Mouse Mutant Archive Repository (CD4-Cre), and used at 8–12 weeks of age. CD4-Cre and p110α^flox/flox^ mice were crossed to obtain CD4-Cre^+/−^ p110α^flox/flox^ (p110α^−/−^ΔT) mice, and these crossed with CD4-Cre^−/−^ p110α^flox/flox^ (WT) mice to obtain p110α^−/−^ΔT and WT littermates used in the experiments. All experimental procedures were performed according to established institutional and national guidelines under project licenses PROEX 181/15 (JMR, CIB) and PROEX 330/15 (PP, ISCIII) from the Consejeria de Medio Ambiente y Ordenación del Territorio de la Comunidad de Madrid. Mice were genotyped for Cre, p110α, p110αflox, or Cre-mediated removal of floxed p110α using the oligonucleotides and conditions described in Ref. (24).

### Antibodies, Inhibitors, and Other Reagents

Antibodies used were monoclonal rat anti-mouse CD3ε [YCD3-1 (44)]; rat anti-mouse CD4 [GK1.5 (45)]; rat anti-mouse CD8 [53–6.72 (46)]; syrian hamster anti-mouse CD28 [37.51 (47)]; rat anti-mouse CD44 (IM-7.8.1); rat anti-mouse CD62L (MEL-14); armenian hamster anti- ICOS (CD278) [C398.4A (48, 49)]; anti-IL-4 (11B11); and anti-IFN-γ (XMG.1). They were prepared in-house as protein A- or protein G-purified preparations from hybridoma supernatants, or purchased from eBioscience (37.51). Where needed, they were coupled with biotin, FITC, or DyLight_647_ in our laboratory using standard methods or as recommended by the manufacturer. Anti-γδ-FITC was from BD Biosciences, RM-5 anti-CD4-FITC, -PE, -PE-Cy7, and -APC conjugates, PE conjugates of anti-mouse CD8a, anti-CD19, anti-CD25, anti-NK1.1, anti-T-bet (4B10), and anti-Foxp3 (FJK-16s) antibodies; APC conjugates of anti-CD62L-, anti- ICOS- (C398.4A), anti-PD-1- (J43) antibodies were from eBioscience. Anti-mouse CXCR5-Biotin (2G8) was from BD-Pharmingen. Anti-CD107a (LAMP-1) (eBio1D4B) Alexa Fluor 488 conjugate and anti-Granzyme B-PE (NGZB-PE) were from eBioscience; anti-Granzyme A-FITC (3G8.5-FITC) was from Santa Cruz Biotechnology.

Anti-phosphotyrosine antibody (PY-20) was from GE Healthcare. Polyclonal rabbit anti-ZAP-70 and anti-CD4 antibodies were obtained and purified as described previously (12, 50). Rabbit antibody specific for dually phosphorylated ERK (Anti-Active MAPK ref. V6671) was from Promega; rabbit anti-phospho-p38 MAPK (Thr180/Tyr182) (#9211) and monoclonal rabbit anti-phospho-Akt(Ser473) (D9E, #4060) antibodies were from Cell Signaling Technology. Conjugates of Streptavidin with AlexaFluor647 (Molecular Probes), PE (Southern Biotech) and PE-Cy7 (BD-Pharmingen) were also used. Peanut agglutinin FITC conjugate was from Vector.

Horseradish peroxidase-coupled polyclonal goat antibodies specific for mouse IgM, IgG1, IgG2b, IgG2c, and IgG3 immunoglobulin were from Southern Biotech.

Rabbit polyclonal anti-ERK antibody was from Upstate Biotechnology. Rabbit anti-PI3K p110δ (H-219) was from Santa Cruz Biotechnology; the rabbit anti-p110α monoclonal antibody C73F8 was from Cell Signaling Technology.

Recombinant human IL-2 and mouse IL-4 and IL-6 were from Preprotech; recombinant human TGF-β was from R&D. PI3Kα inhibitor (A66) was from Selleckchem; the PI3K PI3Kδ Inhibitor IC87114 was from Symansis Pty. The P38 inhibitor SB203580 was from Sigma; the MEK1 inhibitor U0126 was from Calbiochem.

### Cell Separation

Spleen or thymus cell suspensions were prepared in culture medium (Click’s medium supplemented with 10% heat inactivated fetal bovine serum) and passed through 30-µm filters to remove clumps. Naive CD4^+^ (CD4^+^CD62L^+^) T cells were isolated using mouse naive CD4^+^ T cell isolation kit II (130-090-860, Miltenyi Biotech, Bergisch Gladbach, Germany). CD8^+^CD62L^+^ T cells were obtained with the CD8a T Cell Isolation Kit (130-104-075) and anti-CD62L beads (Miltenyi Biotech). Where required, CD4^+^ T cells were purified to >98% CD4^+^ T cells by sorting using a FACS Vantage-DIVA flow cytometer (B-D Bioscience).

### Cell Activation and Differentiation

CD4^+^CD62L^+^ or CD8^+^CD62L^+^ T cells were cultured at 0.5–1 × 10^6^ cells/ml in 24-well culture plates (Costar 3524, 1 ml/well) or 96-well culture plates (Costar 3590, 0.2 ml/well) previously coated with anti-CD3 (YCD3-1, 5–10 µg/ml) plus soluble anti-CD28 (2.5 µg/ml), as described in the Section “Results.” At 72 h, supernatants were taken for cytokine determination. Differentiation of naive CD4^+^ T cells into distinct functional effector subsets was performed by activation with anti-CD3 and anti-CD28 antibodies plus cytokines and antibodies as follows: for Th1 differentiation, 20 ng/ml IL-12 and 5 µg/ml anti-IL-4 (11B11); for Th2 differentiation, 10 ng/ml IL-4 and 10 µg/ml anti-IFN-γ (XMG.1); for Th17 differentiation, 20 ng/ml IL-6, 5 ng/ml human TGF-β1 plus anti-IL-4 and anti-IFN-γ; for Tfh differentiation, cells were cultured in the presence of anti-IL-4 and anti-IFN-γ antibodies plus 20 ng/ml IL-6. Treg differentiation from naive CD4^+^ T cells was achieved with anti-CD3 (YCD3-1, 5 µg/ml) plus huIL-2 (20 ng/ml), 5 ng/ml TGF-β1, 5 µg/ml anti-IL-4, and 10 µg/ml anti-IFN-γ. At 48 h, cells were stained for Foxp3 with the PE anti-mouse/rat FoxP3 staining set (eBioscience), according to the manufacturer’s instructions.

Where needed, CD8^+^CD62L^+^ T cells were first activated for 72 h with anti-CD3 plus anti-CD28, washed, adjusted to 15 × 10^4^ cells/ml in culture medium and then expanded for further 96 h in the presence of 20 ng/ml huIL-2.

### Elongation Assays

Naive CD4^+^ cells were first differentiated into Tfh cells that express high levels of ICOS. Elongation assays were performed in 24-well culture plates containing glass coverslips previously coated with anti-ICOS antibody or Poly-l-Lysine (10 µg/ml in PBS), as described (20). After washing with PBS, the wells received 2 × 10^5^ cells in 0.5 ml of PBS, 10 mM HEPES, 0.1% glucose, pH 7.2. The plates were briefly spun and then incubated for 60 min at 37°C, washed twice with PBS, and fixed with 4% formaldehyde 5 min at 37°C. After washing with PBS, the coverslips were mounted on Vectastain (Vector). The cells were analyzed in an Axioplan Universal microscope (Carl Zeiss, Jena, Germany) and a Leica DFC 350 FX CCD at a 40× magnification to acquire images. Cell elongation was expressed as the Axis Ratio of the major and minor axis of the ellipse fitting the cell using the shape descriptors of the ImageJ 1.51j8 public domain software (National Institutes of Health).

### Cytokine Detection

Cytokines in culture supernatants (IL-2, IL-4, IL-10, IL-17A, IL-21, IFN-γ, and TNF-α) were determined with Ready-SET-Go! kits (eBioscience).

### Immunoblot Analysis

Phosphorylated Tyr in proteins and activated Erk and P38 MAP kinases were determined in lysates of activated cells. Freshly isolated CD4^+^CD62L^+^ T cells from the spleen of WT or p110α^−/−^ΔT mice were washed with PBS and activated for 20 min at 37°C in serum-free medium by mixing the cells at 50 × 10^6^/ml with an equal number of latex beads coated with anti-CD3 or control antibodies (5 µg/ml) in the presence of anti-CD28 antibodies (5 µg/ml), as described in Ref. (12). Separation of cell lysates by SDS-PAGE, transfer to PVDF membranes, and immunoblot was performed as described previously in detail (12, 51). ImageJ 1.51j8 was used for densitometry analysis of immunoblots.

### Intracellular Staining and Flow Cytometry

For staining of the transcription factors Foxp3 and T-bet, or Granzymes A and B, the cells were cultured for the times specified in each case. After washing, they were fixed, permeabilized, and stained with fluorochrome-conjugated antibodies using the Transcription Factor Staining Buffer Set (Affimetrix/eBioscience), according to the manufacturer’s instructions. Staining of CD107a was performed as described in detail in Ref. (52). Flow cytometry analysis of surface and intracellular stained cells was performed in a FC-500 flow cytometer (Beckman Coulter, Brea, SA, USA).

### Immunization with KLH

Keyhole limpet hemocyanin (Sigma) was injected at 100 μg/mouse. It was either emulsified v:v in Freund’s complete adjuvant (FCA, Sigma-Aldrich) and injected s.c. in two sites in the base of the tail, or mixed with Alum (Imject Alum, Thermo Scientific) and injected i.p. Mice were bled and sacrificed on day 7 (FCA) or day 10 (Alum) after immunization. Cells from inguinal lymph nodes (FCA) or the spleen (Alum) of individual mice were analyzed by flow cytometry, or cultured in round-bottom 5 ml polystyrene tubes at 5 × 10^6^ cells/ml plus 100 µg/ml KLH in 1 ml of culture medium. Culture supernatants were taken at 96 h to determine KLH-induced cytokine secretion. The titer of anti-KLH-specific antibodies of the IgM, IgG1, IgG2b, IgG2c, and IgG3 subclasses were determined in the serum of individual mice, as described previously (51).

### Melanoma Cell Growth and Melanoma-Specific Responses

Mouse melanoma B16.F10 cells from a mycoplasma-free stock were maintained by serial passage in culture medium. Primary B16.F10 tumors were induced by s.c. inoculation of 10^5^ B16.F10 melanoma cells in one flank of mice. Tumor size was periodically measured with calipers by experimenters blind to phenotype. Mice were sacrificed when tumor diameter reached 10 mm, or on day 21 after inoculation, and spleen was obtained.

Spleen cells from individual mice were analyzed by flow cytometry, or cultured (5 × 10^6^ cells/culture) in 1 ml of culture medium in round-bottom 5 ml polystyrene tubes containing 100 µg/ml of protein extract from B16.F10 cell lysates. Supernatants were collected after 6 days of culture and cytokine content determined by ELISA.

### Statistical Analyses

The statistical analyses were performed with GraphPad Prism 6.05 using the Student’s *t*-test, or the log-rank Mantel–Cox test. Significant differences between data are indicated by asterisks (*p < 0.05, **p < 0.01, ***p < 0.001).

## Ethics Statement

All experimental procedures were performed according to established institutional, local, and national guidelines. The studies were first approved by the Animal Welfare Commitees of the Centro de Investigaciones Biológicas (OEBA-CIB) and the Centro Nacional de Microbiología (OEBA-Majadahonda). Then, the study was approved by the Ethics Commitee, Subcommiteee of Bioethics, of the Consejo Superior de Investigaciones Científicas as well as by the Ethics and Animal Welfare Commitee of the Instituto de Salud Carlos III (CEYBA). The study was eventually authorized by the “Consejeria de Medio Ambiente y Ordenación del Territorio de la Comunidad de Madrid” under project licenses PROEX 181/15 (to JR) and PROEX 330/15 (to PP).

## Author Contributions

JR, PP, and UD conceived, designed, and supervised the study, and wrote, reviewed, and revised the manuscript. LA-F, GO, MM-C, YA-A, and JR performed the experiments and acquired the data, analyzed and interpreted the results.

## Conflict of Interest Statement

The authors declare that the research was conducted in the absence of any commercial or financial relationships that could be construed as a potential conflict of interest.
