# Supplementary material for: T-Cell-Specific Loss of the PI-3-Kinase p110α Catalytic Subunit Results in Enhanced Cytokine Production and Antitumor Response
Source: Front Immunol. 2018 Feb 27;9:332. doi: 10.3389/fimmu.2018.00332 (PMC5835342; doi:10.3389/fimmu.2018.00332)
Supplement: Supplementary file 1 [file Data_Sheet_1.PDF]

Supplementary Material

T-cell specific loss of the PI-3 kinase p110α catalytic subunit results in enhanced cytokine production and anti tumor response

Laura Aragoneses-Fenoll[1], Gloria Ojeda[1], Maria Montes-Casado[1], Yeny Acosta-Ampudia[2,§], Umberto Dianzani[3], Pilar Portolés[1,*], José M. Rojo[2,*]

[1]Unidad de Inmunología Celular, Centro Nacional de Microbiología, Instituto de Salud Carlos III, Majadahonda, Madrid, Spain

[2]Departamento de Medicina Celular y Molecular, Centro de Investigaciones Biológicas, CSIC, Madrid, Spain

[3]Interdisciplinary Research Center of Autoimmune Diseases (IRCAD) and Department of Health Sciences, University of Piemonte Orientale (UPO), Novara, Italy

[§]Current address: Center for Autoimmune Diseases Research (CREA), School of Medicine and Health Sciences, Universidad del Rosario, Colombia

*** Correspondence:**
Pilar Portolés
pportols@isciii.es
José M. Rojo
jmrojo@cib.csic.es

Supplementary Data

Supplementary Figure 1. Analysis of spleen and thymus populations in WT and p110α$^{-/-}$ΔT mice. **(A)** Cell count and flow cytometry analysis of the spleens from WT and p110α$^{-/-}$ΔT mice. The number of cells/spleen and the percentage of CD4$^+$ and CD8$^+$ T lymphocytes, of γδ T (CD3$^+$γδ$^+$) and NKT (CD16$^+$CD3$^+$) cells, of B lymphocytes (CD19$^+$ cells), or NK cells (CD16$^+$ CD3$^-$ cells) were determined. The percentage of naïve CD4$^+$ T cells (CD4$^+$CD44^{low}CD62L^{high}); memory T cells (CD4$^+$CD44^{high}CD62L^{low}) and Treg cells (CD4$^+$Foxp3$^+$) in the CD4 subset were also determined. Data show the results from individual mice and the mean ±SE. Asterisks indicate significant differences ($p<0.05$) between groups, as determined with the Student´s t test. **(B)** Immunoblot analysis of PI3K p110α expression in lysates of CD4$^+$ T lymphocytes from WT and p110α$^{-/-}$ΔT mice. Expression of the p110δ PI3K catalytic subunit or other surface (CD4) or intracellular (Erk) proteins was analyzed for comparison. **(C)** Flow cytometry analysis of CD4$^+$ and CD8$^+$ expression in thymus cells from WT and p110α$^{-/-}$ΔT mice. Figures inside the histograms represent the percentage of cells in each quadrant.

Supplementary Figure 2. A) PI3-kinase 110α-deficient Th1 and Th17 cells show enhanced secretion of effector cytokines. Naive CD4$^+$ T lymphocytes from WT or p110α-T cell deficient (p110α$^{-/-}$ΔT) mice were differentiated “in vitro” for 1 day (T-bet) or 4 days (cytokines) with anti-CD3 plus anti-CD28 under Th1or Th17 conditions. Then, cells were stained with anti-T-bet and

analyzed by flow cytometry, and culture supernatants were analyzed for cytokine content (IL-2, IFN-γ, or IL-17A), as indicated. **B)** PI3-kinase p110α deficiency enhances effector functions of activated $CD8^+$ T cells. Naive ($CD8^+$ $CD62L^+$) $CD8^+$ T lymphocytes were isolated from the spleen of WT or $p110\alpha^{-/-}\Delta T$ mice. They were activated for three days with anti-CD3 and anti-CD28. Then, $CD8^+$ T cells were cultured for further 72h in the presence of IL-2. Eventually, IFN-γ and TNF-α content in the supernatants was determined. Mean of triplicate cultures ±SE from one experiment of two performed. Significant differences (* $p<0.05$; *** $p<0.001$) are indicated, as determined by the Student´s t test.

Supplementary Figure 3. Effect of T-cell PI3-kinase p110α deficiency on cytokine and antibody responses against the protein antigen KLH. **(A)** WT (n=8) or $p110\alpha^{-/-}\Delta T$ mice (n=7) were immunized s.c. with KLH plus FCA. Serum from individual mice was taken on day seven after immunization, and anti-KLH antibodies of the IgG1, IgG2a, IgG2c, IgG3, and IgM isotypes were determined. The number of inguinal lymph node cells from each mouse is also shown. Data from two experiments. Values from individual mice as well as means ±SE are shown. No significant differences were observed, as determined by the Student´s t test. **(B)** KLH-specific response of WT or $p110\alpha^{-/-}\Delta T$ mice injected i.p. with KLH in Alum. Spleen cells from mice immunized ten days before were cultured "in vitro" for 96h in the presence of KLH, and cytokines in the supernatant (IL-2, IL-17A, TNF-α) were determined. Data from two experiments. Individual values from n=8 (WT) or n=7 ($p110\alpha^{-/-}\Delta T$) mice, as well as means ±SE, are shown. No significant differences were observed, as determined by the Student´s t test. Note that Y scales used in each case were the same as those in Figure 05.

Supplementary Figure 4. Effect of PI3-kinase p110α removal on naive T cell activation. **(A)** Naive $CD4^+$ T lymphocytes from WT or p110α-T cell deficient ($p110\alpha^{-/-}\Delta T$) mice were activated or not with plate-bound anti-CD3 plus anti-CD28, as indicated. At 72 h, cell proliferation was determined by a colorimetric methods using MTT. Mean from three experiments ±SE. **(B)** Naive $CD4^+$ T lymphocytes from WT or p110α-T cell deficient ($p110\alpha^{-/-}\Delta T$) mice were differentiated to Tfh cells for 72h by activation with plate-bound anti-CD3 plus anti-CD28 and IL-6. Then, cells were counted (left panel) and the IL-21 content in the supernatants was determined by ELISA (right panel). Mean from triplicate cultures ±SE. Significant differences were determined using the Student´s t test (*, $p<0.05$).

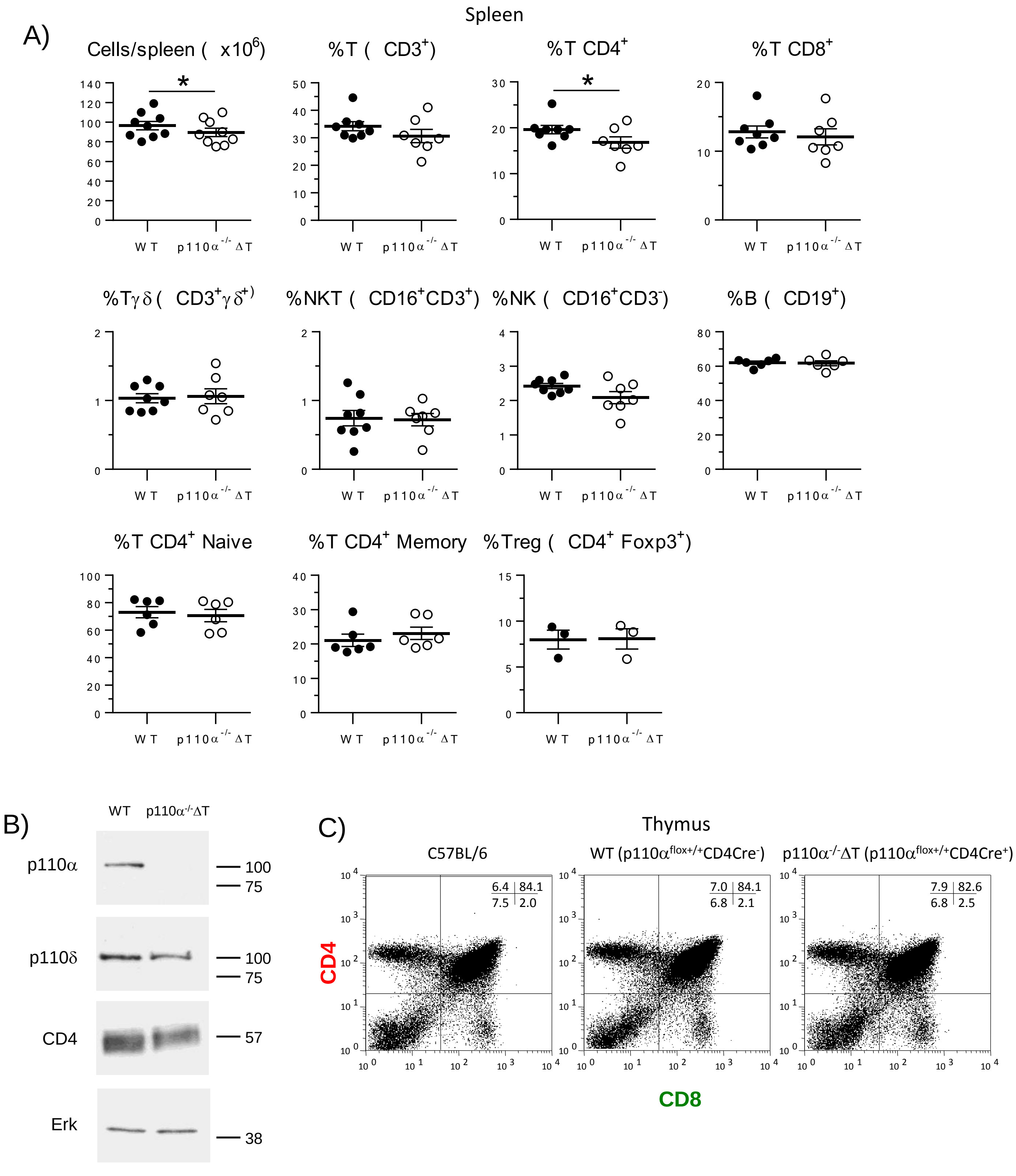

Supplementary Figure S1

A)

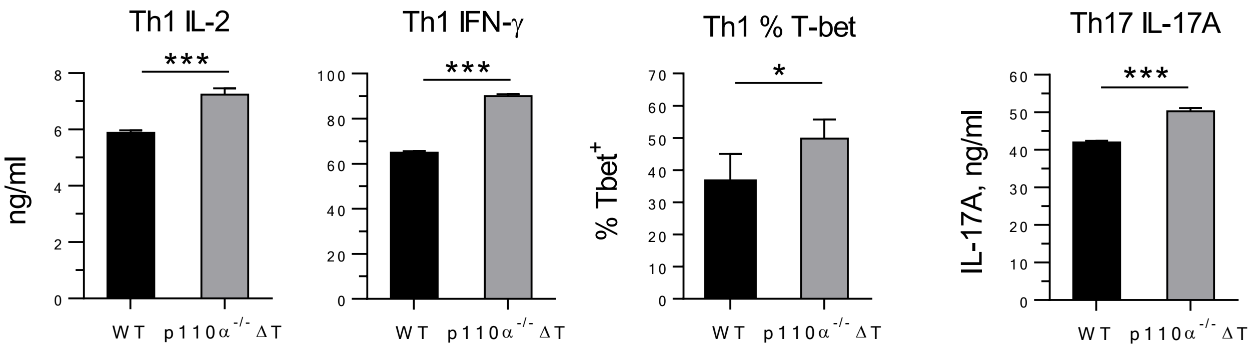

B)

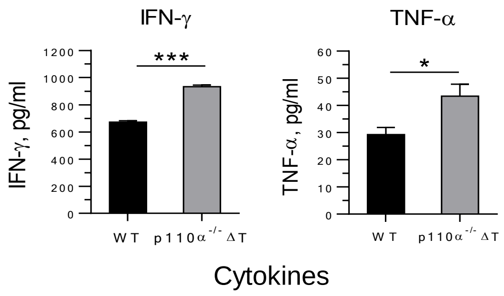

Supplementary Figure S2

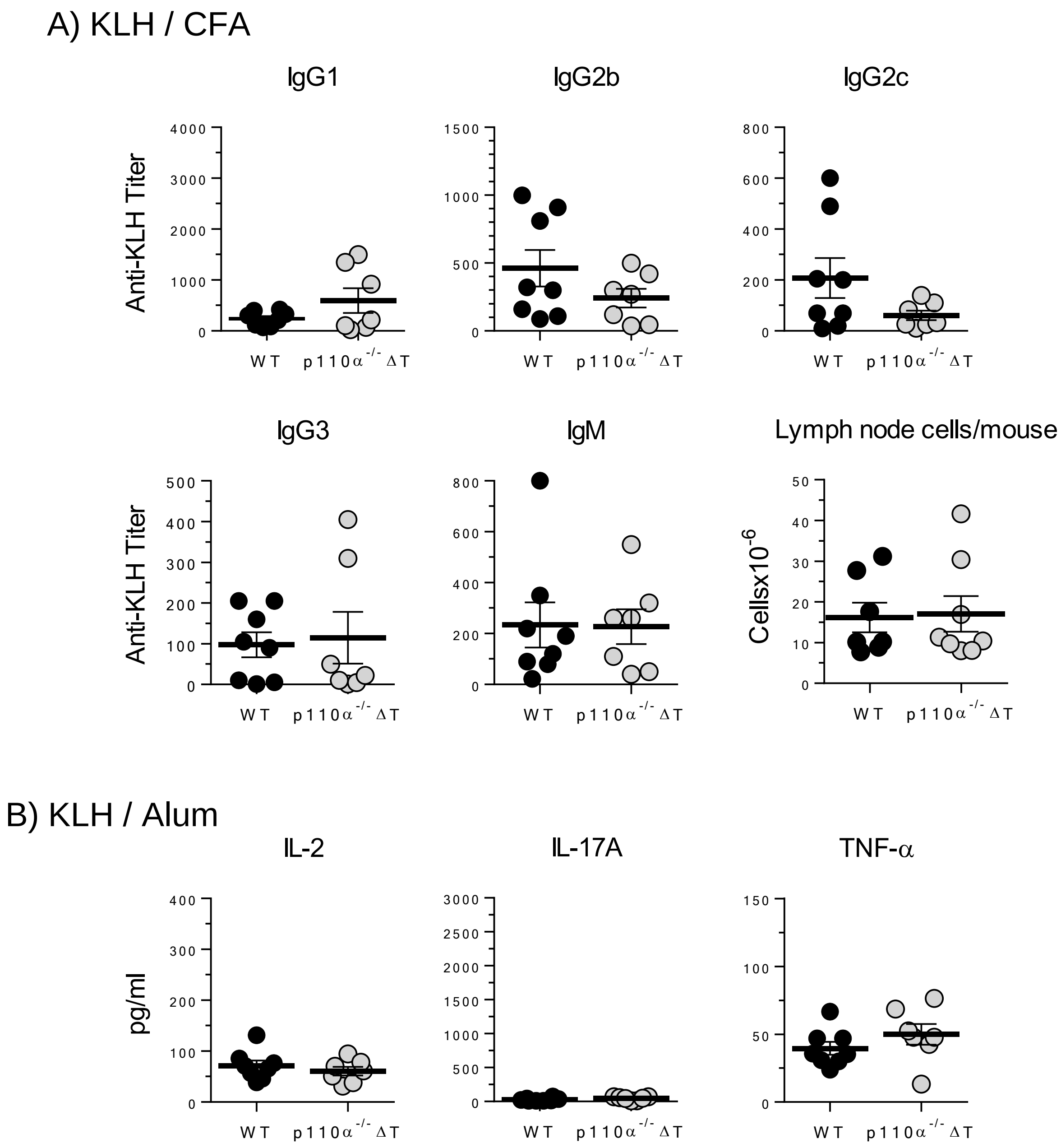

Supplementary Figure S3

A) Th0 proliferation

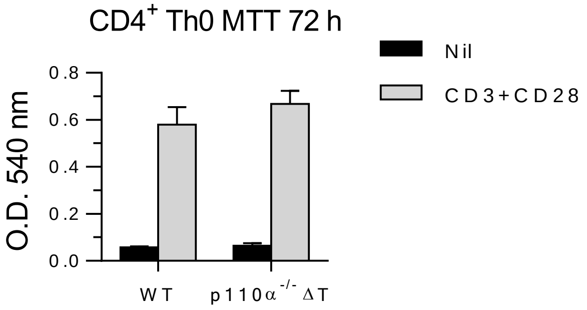

B) Tfh cell number vs IL-21 secretion

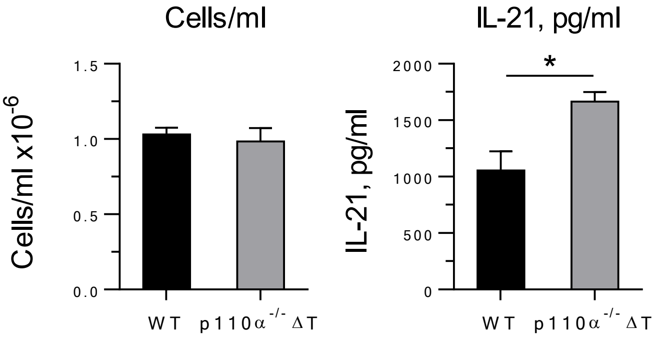

Supplementary Figure S4
